# Comprehensive landscape of immune-based classifier related to early diagnosis and macrophage M1 in spinal cord injury

**DOI:** 10.18632/aging.204548

**Published:** 2023-02-23

**Authors:** Zhao Zhang, Zhijie Zhu, Xuankang Wang, Dong Liu, Xincheng Liu, Zhenzhou Mi, Huiren Tao, Hongbin Fan

**Affiliations:** 1 Department of Orthopaedics, Xi-Jing Hospital, The Fourth Military Medical University, Xi’an 710032, China; 2 Department of Orthopaedics, Shenzhen University General Hospital, Shenzhen 518052, China

**Keywords:** spinal cord injury, immune, machine learning, diagnosis, macrophage

## Abstract

Numerous studies have documented that immune responses are crucial in the pathophysiology of spinal cord injury (SCI). Our study aimed to uncover the function of immune-related genes (IRGs) in SCI. Here, we comprehensively evaluated the transcriptome data of SCI and healthy controls (HC) obtained from the GEO Database integrating bioinformatics and experiments. First, a total of 2067 DEGs were identified between the SCI and HC groups. Functional enrichment analysis revealed substantial immune-related pathways and functions that were abnormally activated in the SCI group. Immune analysis revealed that myeloid immune cells were predominantly upregulated in SCI patients, while a large number of lymphoid immune cells were dramatically downregulated. Subsequently, 51 major IRGs were screened as key genes involved in SCI based on the intersection of the results of WGCNA analysis, DEGs, and IRGs. Based on the expression profiles of these genes, two distinct immune modulation patterns were recognized exhibiting opposite immune characteristics. Moreover, 2 core IRGs (FCER1G and NFATC2) were determined to accurately predict the occurrence of SCI via machine learning. qPCR analysis was used to validate the expression of core IRGs in an external independent cohort. Finally, the expression of these core IRGs was validated by sequencing, WB, and IF analysis *in vivo*. We found that these two core IRGs were closely associated with immune cells and verified the co-localization of FCER1G with macrophage M1 via IF analysis. Our study revealed the key role of immune-related genes in SCI and contributed to a fresh perspective for early diagnosis and treatment of SCI.

## INTRODUCTION

Spinal cord injury (SCI) is a devastating central nervous system (CNS) trauma with severe disabling and fatal rates, resulting in direct or indirect loss of sensory, motor, and other functions below the injury area [1]. The prevalence of SCI is on the rise with about 17,000 new cases each year, and the population is getting increasingly younger [2]. The primary and secondary responses following an injury can trigger a series of destructive cascading phenomena in the spinal cord to further exacerbate nerve cell death and tissue destruction, allowing for poor functional recovery in most patients [3]. At present, the surgical and pharmacological therapies in the clinic still cannot obtain satisfactory results, which brings great psychological and economic burdens to patients and society [4, 5]. Hence, it is extremely crucial to understand the pathological process of SCI deeply to formulate effective therapeutic strategies.

As part of the CNS, the spinal cord has an integral blood-brain barrier that restricts the penetration of immune cells and cytokines from peripheral blood circulation [6]. As such, the spinal cord is widely considered to be an immune-privileged site [7]. However, the integrity of the blood-spinal cord barrier can be disrupted after SCI, resulting in dramatic alterations in the cellular microenvironment of the spinal cord. A substantial population of peripheral immune cells infiltrate the injured area uncontrollably and interacts with glial cells to activate the immune response with the release of extensive inflammatory factors, which exacerbate local tissue damage and recovery [8, 9]. The restoration and recovery of nerves after SCI are frequently intimately connected with an appropriate microenvironment [10]. Under normal conditions, peripheral immune responses are essential for maintaining the homeostasis of the spinal microenvironment. While SCI will directly impair the vegetative innervation function of endocrine and lymphoid organs, causing a prolonged and overactive immune response, which in turn affected the pathophysiological changes at the site of injury [11]. The immune inflammatory response has been recognized as central to the mechanisms of secondary SCI onset and holds an integral role in activating and coordinating other secondary injury mechanisms [12]. A variety of immune cells are involved in the inflammatory response after SCI, including neutrophils, macrophages, B lymphocytes, and T lymphocytes, which can remain at the site of injury long and exert different functions in response to the injury phases and altered signaling in the microenvironment [13, 14]. An increasing number of studies have demonstrated that the inflammatory microenvironment formed from immune cells and cytokines has a dual role in the regenerative and repair processes in SCI [15]. For instance, macrophages are capable of forming both a pro-inflammatory response in the injured spinal cord to exacerbate tissue damage and an anti-inflammatory signal to protect neuronal cells [16]. However, the involvement of immune-related genes (IRGs) in SCI remains unclear.

Recently, bioinformatics analysis combined with machine learning has been widely applied to genomic biodata to detect reliable and robust biomarkers for the diagnosis and treatment of diseases [17, 18]. In this study, we utilized bioinformatics analysis to synthesize the role of IRGs based on peripheral blood transcriptomic data from SCI patients. Subsequently, machine learning was performed to identify 2 immune modulation patterns for SCI patients and screen robust biomarkers to diagnose SCI patients. Finally, we validated these biomarkers with *in vivo* experiments. Our findings provided a solid theoretical foundation for further characterizing the role of immune response in SCI.

## RESULTS

### Identification of DEGs and enrichment analysis in SCI

**Figure 1:**
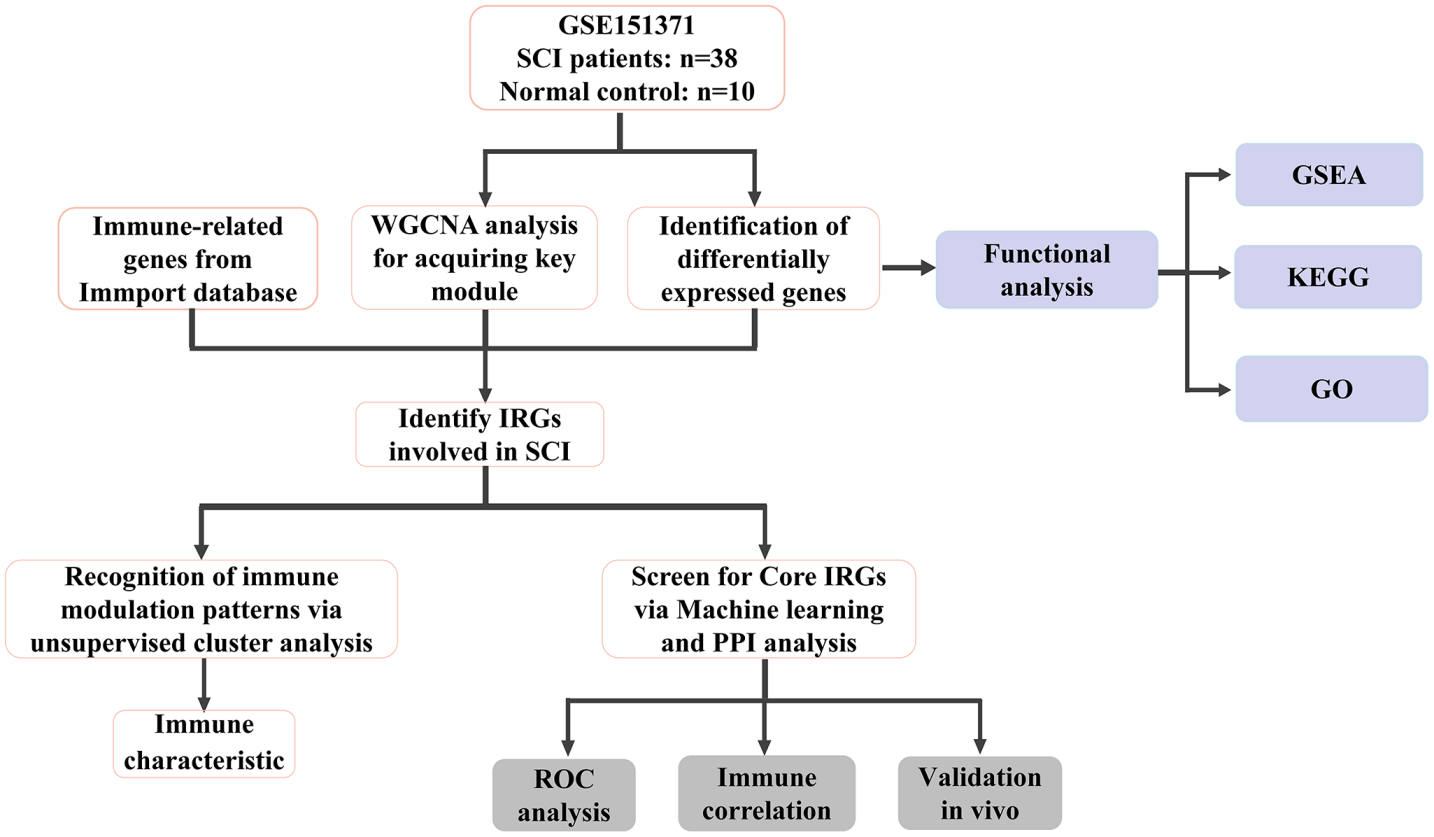
Flow chart of this study.

**Figure 2:**
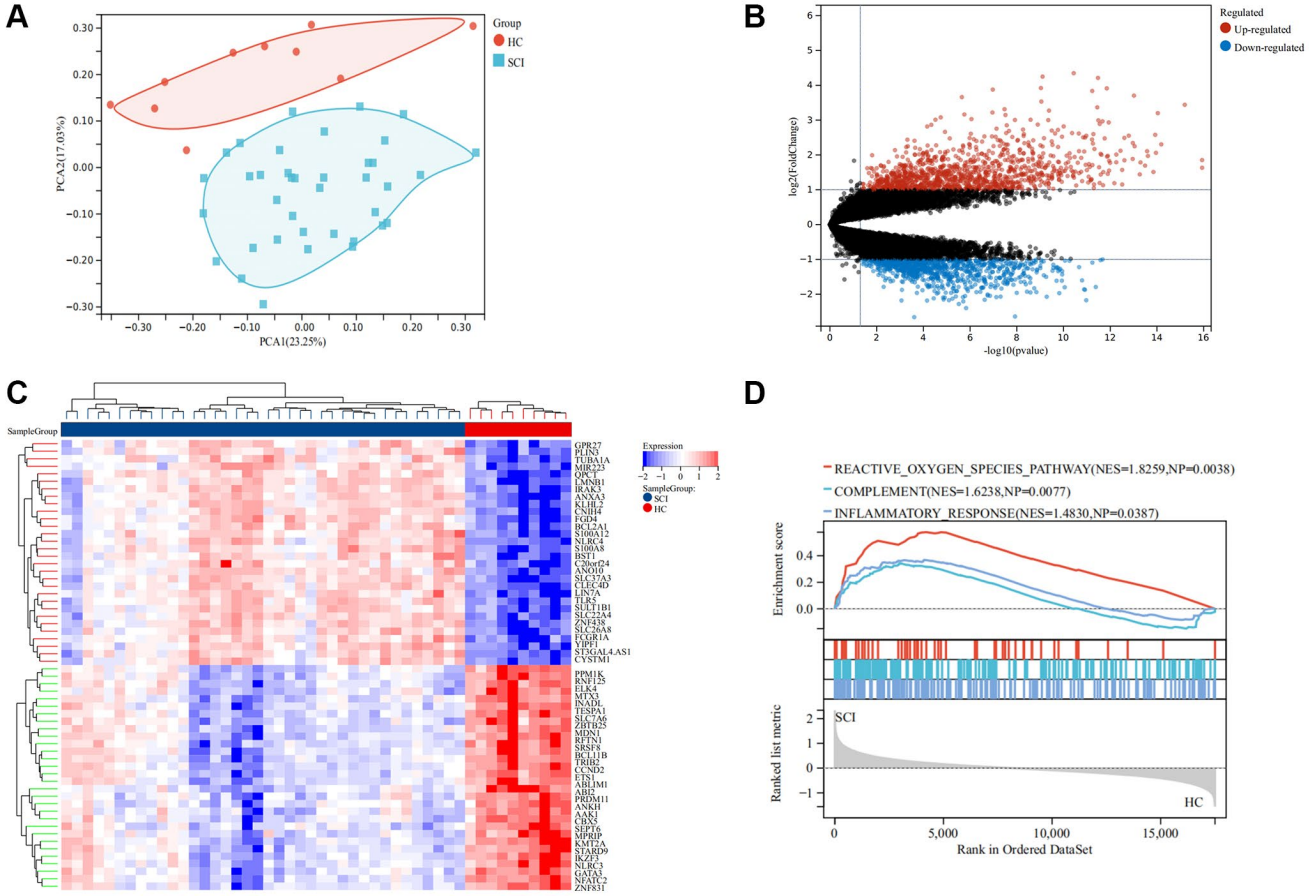
**Screening for differentially expressed genes (DEGs) in SCI.** (**A**) PCA analysis between the SCI and HC groups; (**B**) Volcano map of DEGs, red represent up-regulated genes and blue represent down-regulated genes; (**C**) Volcano map showing DEGs; (**D**) GSEA analysis between the SCI and HC groups.

**Figure 3:**
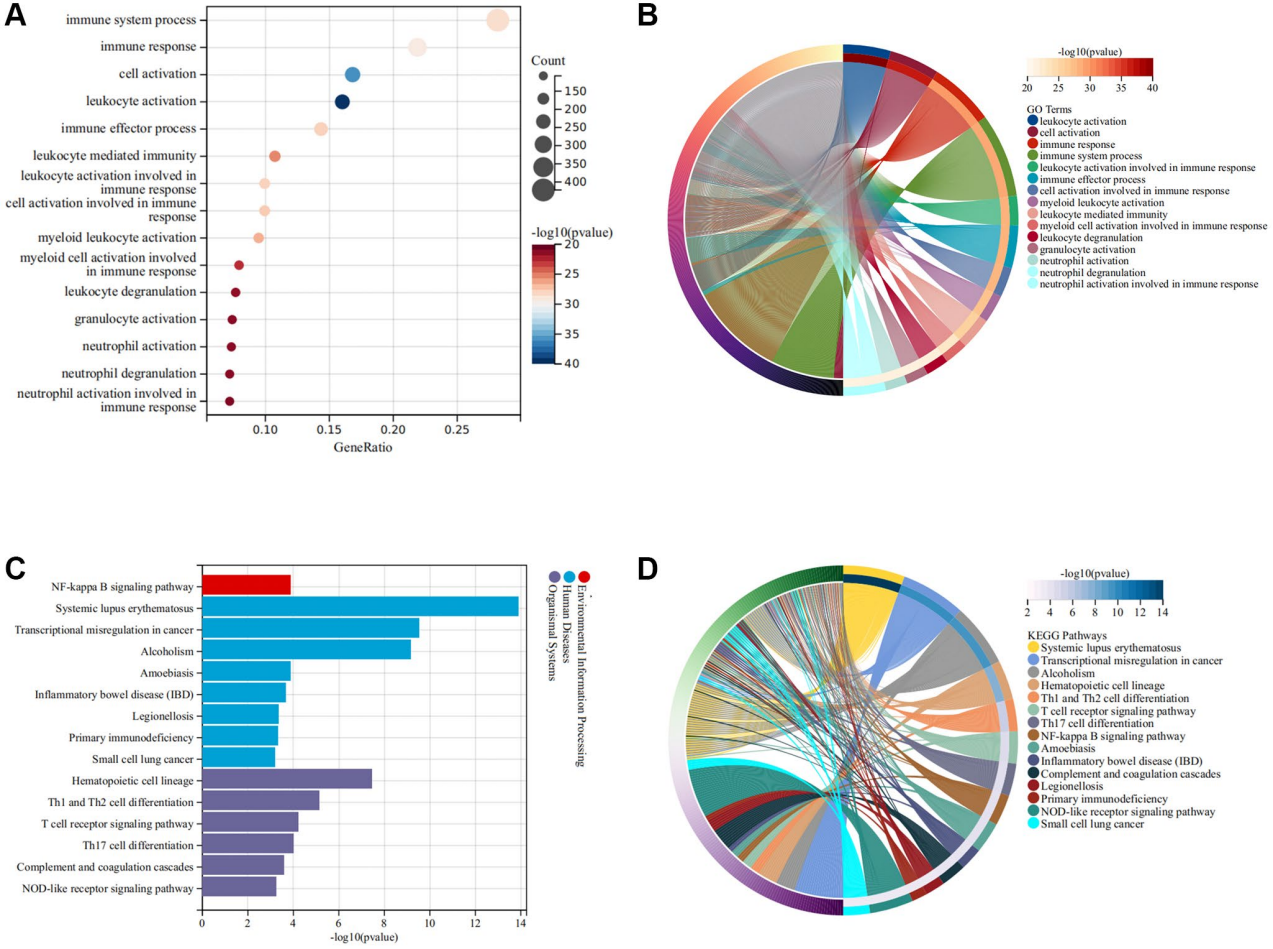
**Functional enrichment analysis of DEGs.** (**A**, **B**) GO terms of DEGs; (**C**, **D**) KEGG pathway of DEGs.

The flow chart of this study was shown in Figure 1. As illustrated in Figure 2A, the SCI and HC groups could be distinguished. Subsequently, a total of 2067 differentially expressed genes (DEGs) were identified between SCI and healthy controls (HC), of which 1160 were up-regulated genes and 907 were down-regulated genes (Figure 2B). Heat map showing the expression of the top 30 DEGs between SCI and HC groups (Figure 2C). Moreover, Gene set enrichment analysis (GSEA) revealed marked activation of inflammatory responses, complement, and reactive oxygen species (ROS) in the SCI compared to the HC group (Figure 2D). Gene Ontology (GO) analysis indicated that these DEGs were concerned with the immune system process, immune response, cell activation, and so on (Figure 3A, 3B). Kyoto Encyclopedia of Genes and Genomes (KEGG) analysis identified that these DEGs were mainly involved in hematopoietic cell lineage, Th1 and Th2 cell differentiation, T cell receptor signaling pathways, etc., (Figure 3C, 3D).

### Evaluation of the immune microenvironment in SCI

**Figure 4:**
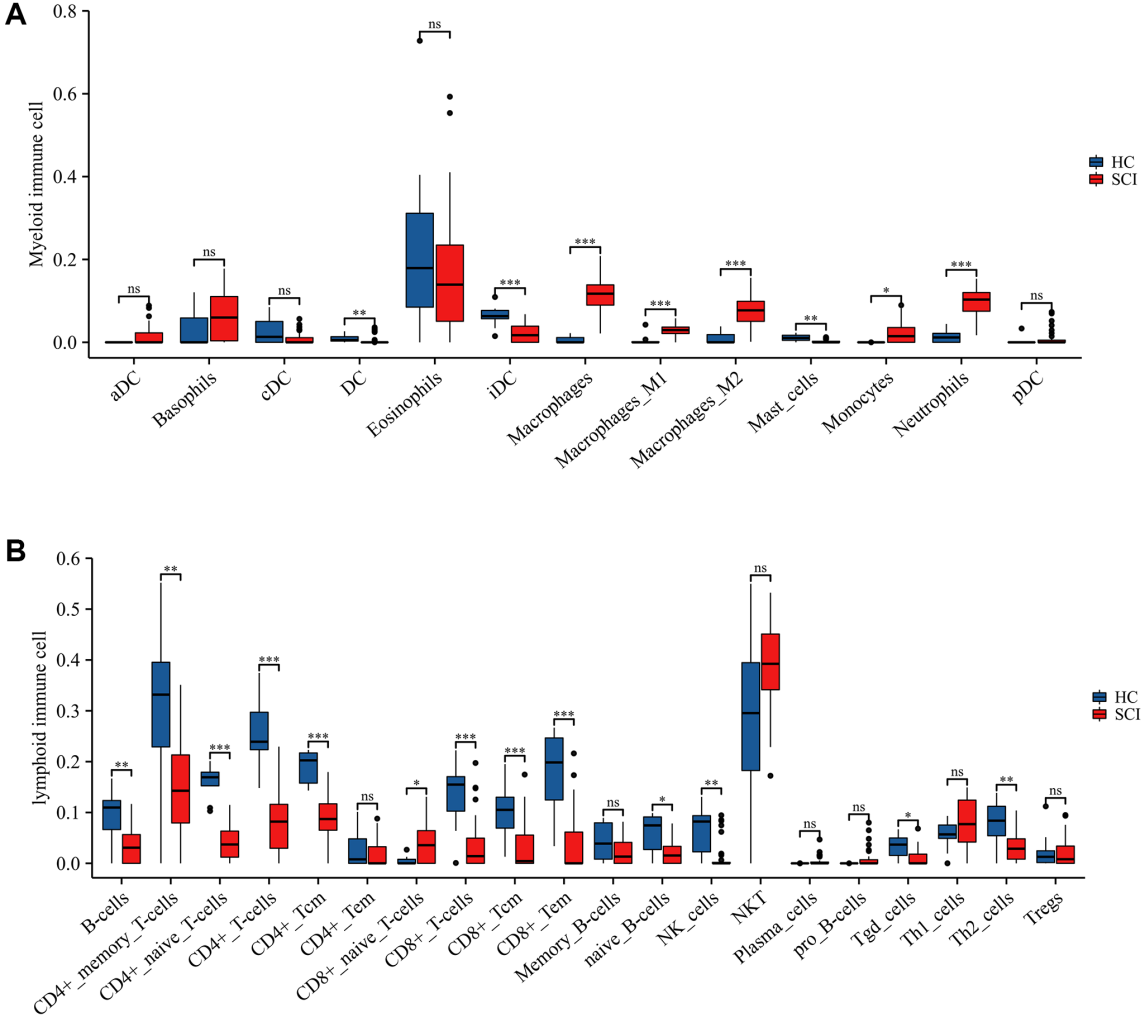
**Immune landscape between SCI and HC groups assessed using xCELL algorithm.** (**A**) Differences in myeloid immune cells; (**B**) Differences in lymphoid immune cells. ^*^p < 0.05; ^**^p < 0.01; and ^***^p < 0.001.

To elucidate the immune microenvironment in peripheral blood after SCI, we applied the xCELL algorithm to assess the abundance of immune infiltrating cells. The results revealed that the expression of macrophages, macrophage M1, macrophage M2, neutrophils, etc., was significantly higher in the SCI group than in the HC group (Figure 4A). On the contrary, the expression of B cells, CD4+ T cells, CD8+ T cells, NK cells, etc. was significantly lower in the SCI group than in the HC group. These findings suggest that immune cell dysregulation was intimately associated with the development of SCI (Figure 4B).

### Screening of key modules and IRGs via WGCNA

**Figure 5:**
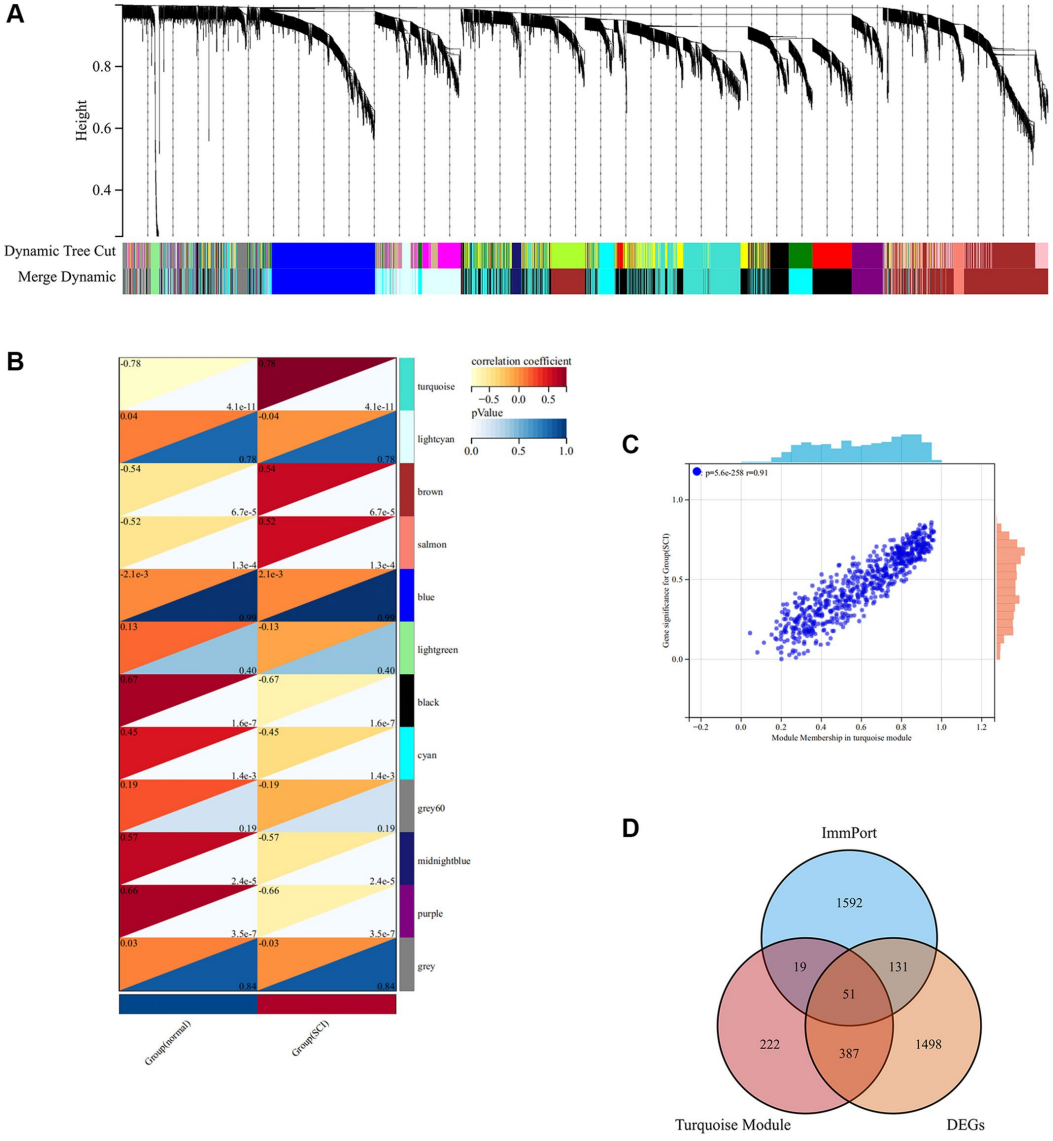
**WGCNA analysis screening key module in SCI.** (**A**) The cluster dendrogram of co-expression genes in SCI; (**B**) Co-relationship of different modules and characteristics; (**C**) Scatter plot showing the correlation between turquoise module and SCI; (**D**) Wayne analysis screening for important IRGs.

Weighted gene co-expression network analysis (WGCNA) was constructed to screen the most relevant module for the onset of SCI. As shown in Figure 5A, a total of 12 modules were identified by hierarchical clustering and dynamic tree-cutting algorithms. Among the 12 modules, we observed that the turquoise module presented the most positive correlation with SCI (Figure 5B). The turquoise module includes a total of 679 genes which may be critical for the pathological progression of SCI (Figure 5C). Finally, a total of 51 major IRGs were identified based on the intersection of DEGs, turquoise modules, and IRGs (Figure 5D).

### Recognition of immune modulation patterns in SCI patients

**Figure 6:**
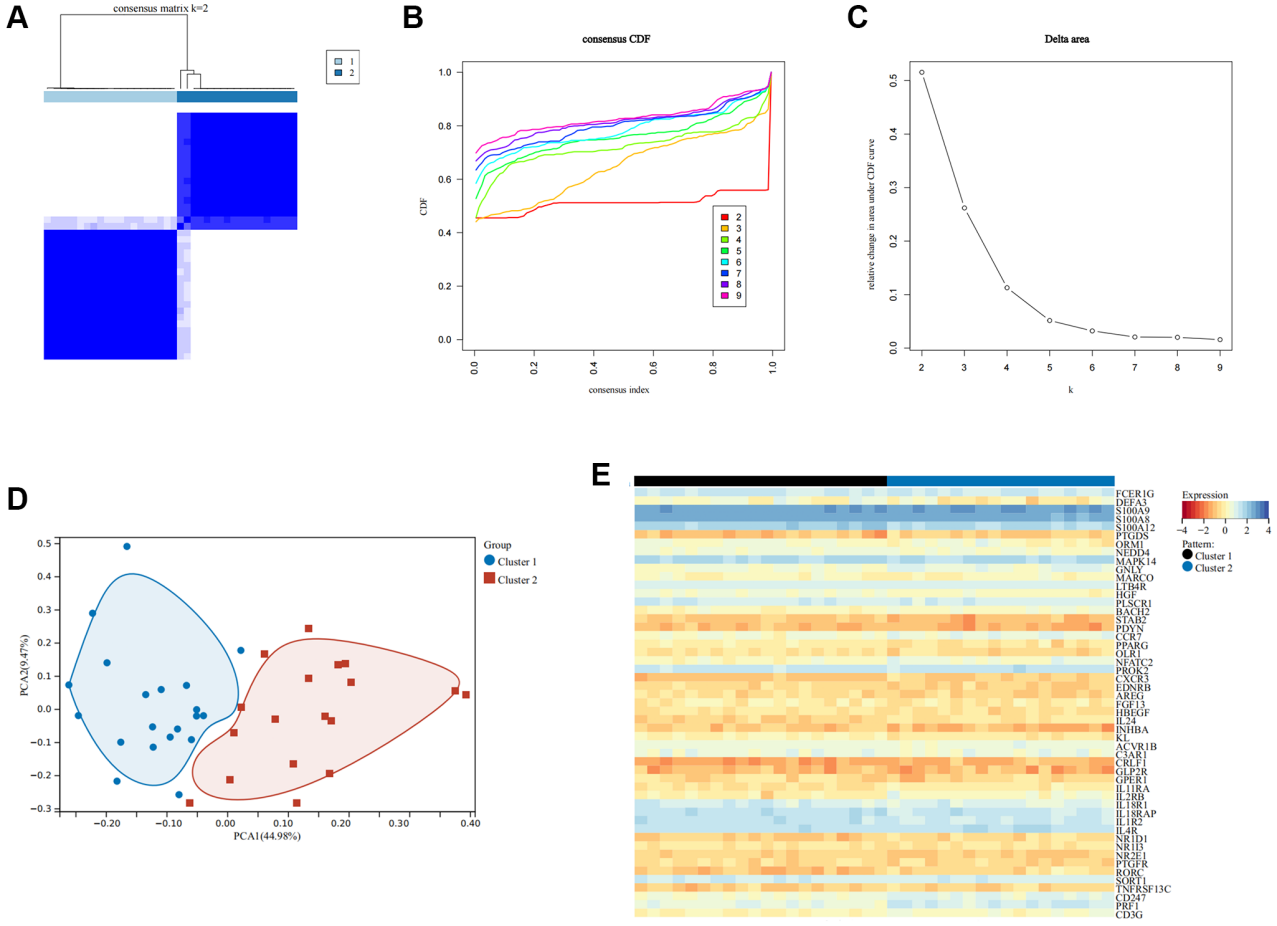
**Recognition of immune modulation patterns in SCI.** (**A**–**C**) Clustering matrix plot at k = 2 via unsupervised clustering analysis; (**D**) PCA analysis of immune modulation patterns; (**E**) Heat map showing the expression of important IRGs.

**Figure 7:**
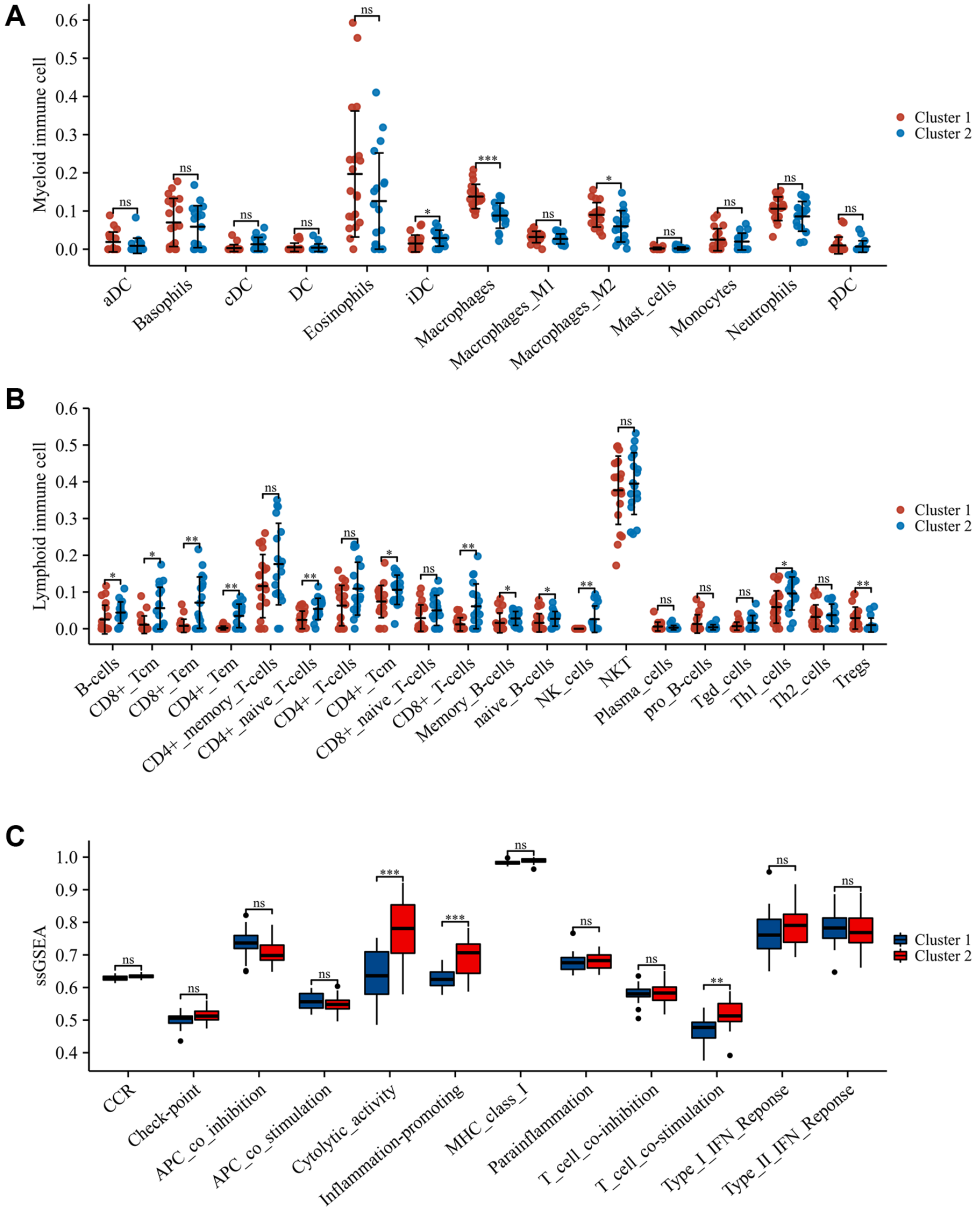
**Immune landscape in different immune modulation patterns.** (**A**) Differences in myeloid immune cells; (**B**) Differences in lymphoid immune cells; (**C**) Differences in immune function. ^*^p < 0.05; ^**^p < 0.01; and ^***^p < 0.001.

To ascertain the immune subtypes in SCI, unsupervised clustering analysis was applied to precisely classify SCI patients based on the expression profiles of 51 IRGs. Eventually, k = 2 was identified as the best number of patterns (Figure 6A–6C). Principal component analysis (PCA) revealed that these two patterns could be distinguished (Figure 6D). The heat map demonstrated the expression of these 51 IRGs in different immune modulation patterns (Figure 6E). xCELL analysis revealed an increased abundance of macrophages, macrophage M2, and Treg infiltration in Cluster1 compared to Cluster 2, while CD4+ T cells, CD8+ T cells, NK cells, etc., showed the opposite results (Figure 7A, 7B). Immune function analysis reveals that inflammation-promoting, T-cell co-stimulation, and cytolytic activity were more pronounced in Cluster 2 (Figure 7C). These results indicated that SCI patients can be divided into immunoreactive and immunosuppressive patterns according to immune status.

### Selection of core IRGs by machine learning and PPI analysis

**Figure 8:**
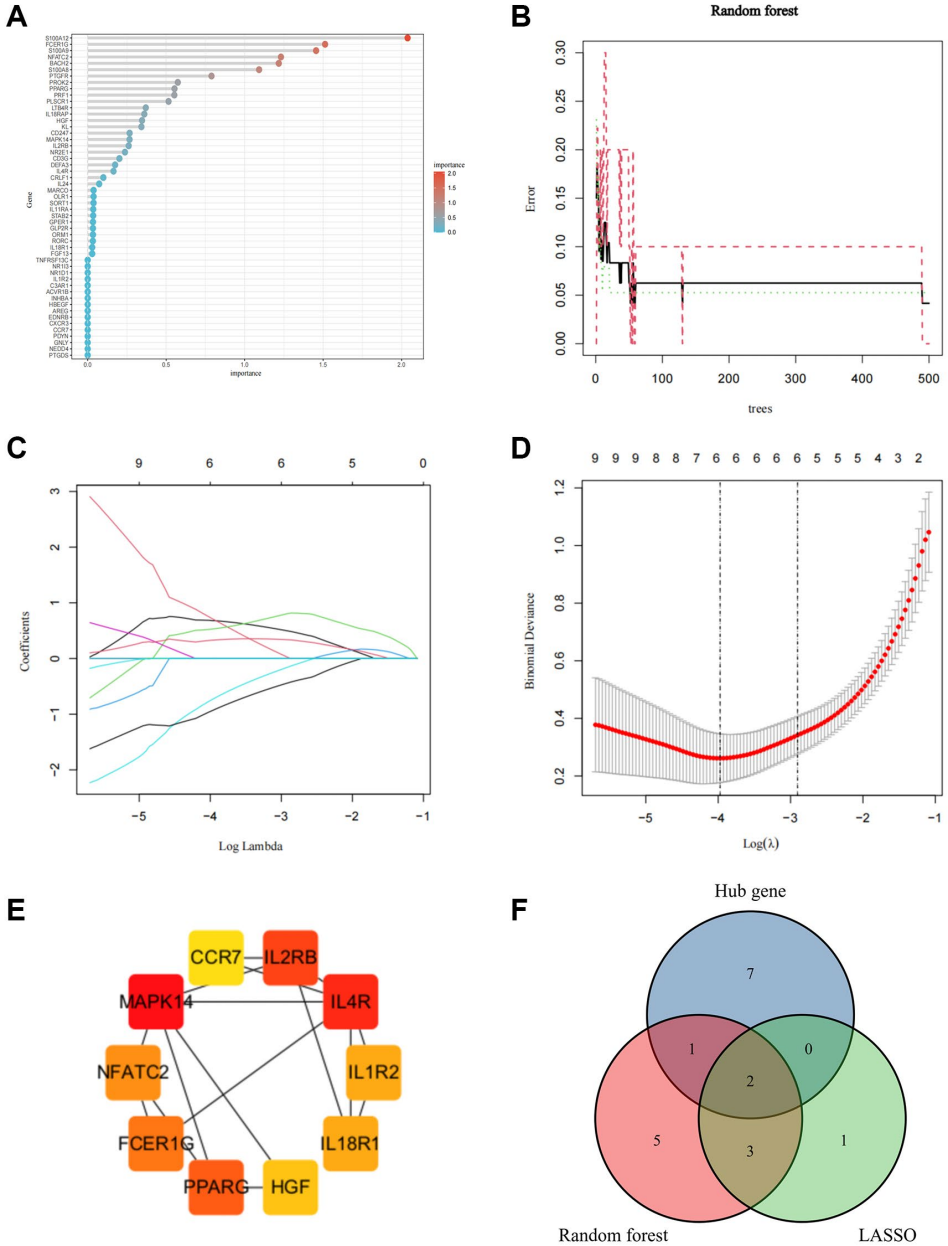
**Screen for Core IRGs in SCI.** (**A**, **B**) Random forest algorithm to screen Core IRGs; (**C**, **D**) LASSO algorithm to screen Core IRGs; (**E**) PPI analysis to screen Core IRGs in Cytohubba; (**F**) Wayne diagram to obtain the intersection of the three algorithms.

To identify stable and reliable biomarkers to diagnose SCI, two machine learning and PPI analyses were used to further screen 51 IRGs. The Random Forest algorithm identified 11 candidate genes with a relative importance score >0.5 (Figure 8A, 8B). The least absolute shrinkage and selection operator (LASSO) algorithm confirmed 6 candidate genes with the minimum lambda value (Figure 8C, 8D). Moreover, the Protein-Protein interaction (PPI) network identified 10 key genes involved in the occurrence of SCI (Figure 8E). Finally, 2 Core IRGs were identified based on the intersection of the results of the above 3 algorithms which were considered key targets for the diagnosis and treatment of SCI (Figure 8F).

### Diagnostic efficacy and verification of core IRGs

**Figure 9:**
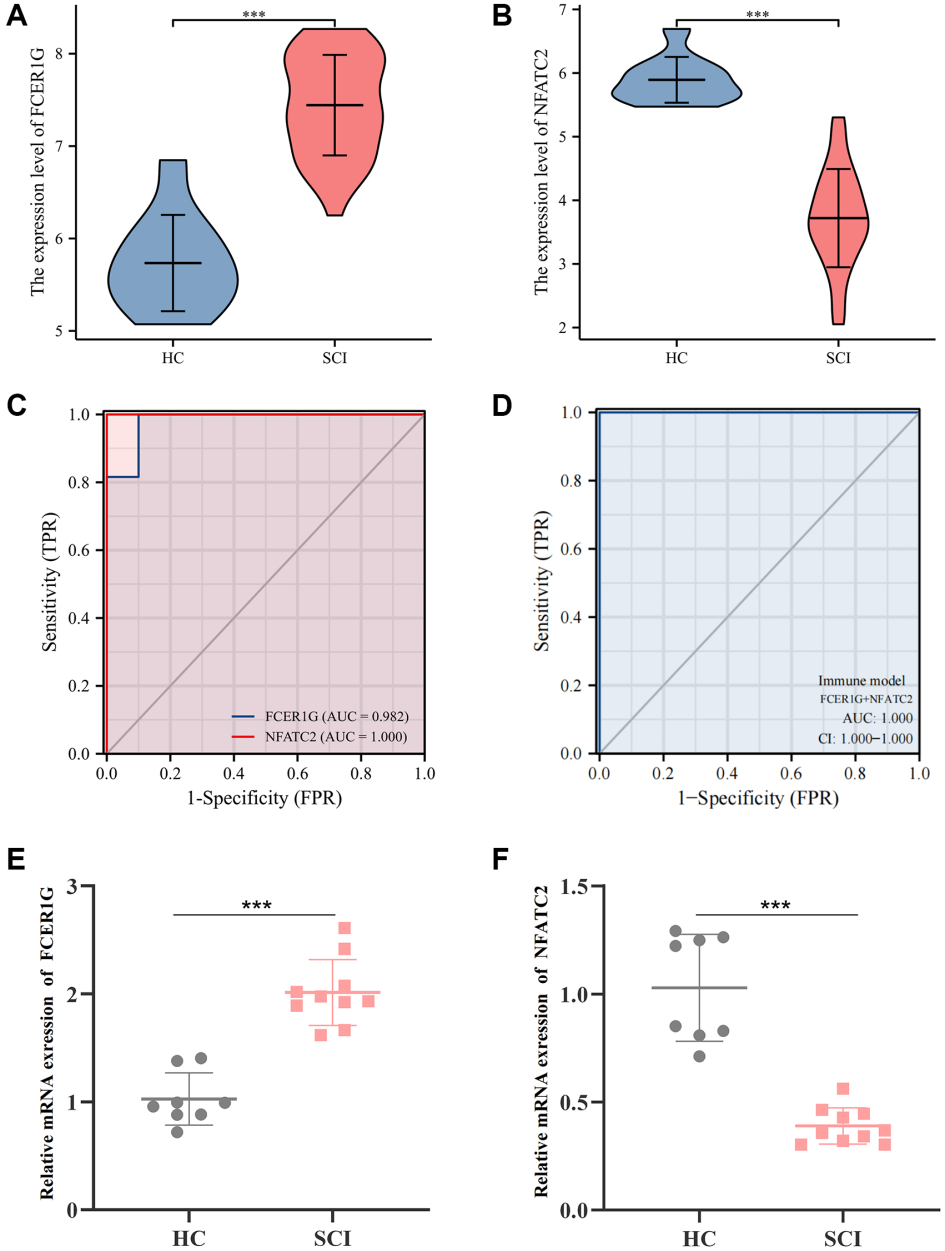
**Diagnostic value and validation of Core IRGs.** (**A**, **B**) The expression levels of Core IRGs in GSE151371; (**C**, **D**) ROC curve analysis for Core IRGs; (**E**, **F**) Validation of Core IRGs expression by qPCR analysis between SCI patients (*n* = 10) and healthy controls (*n* = 8). ^*^p < 0.05; ^**^p < 0.01; and ^***^p < 0.001.

We found that the expression of FCER1G was higher in the SCI group than in the HC group in GSE151371, while NFATC2 was significantly downregulated (Figure 9A, 9B). The receiver operating characteristic (ROC) curve found that the area under curve (AUC) values of FCER1G and NFATC2 were 0.982 and 1.000, respectively (Figure 9C). When fitting these data to one variable, the AUC value of the ROC curve was 1.000, indicating excellent performance in predicting the occurrence of SCI (Figure 9D). Subsequently, quantitative polymerase chain reaction (qPCR) analysis was performed to further assess the expression of Core IRGs in the external validation cohort. The results showed that, compared with the HC group, the expression of FCER1G was elevated in the SCI group, while the expression of NFATC2 was decreased, in agreement with the results of the bioinformatics analysis (Figure 9E, 9F).

### Validation of the core IRGs *in vivo*

**Figure 10:**
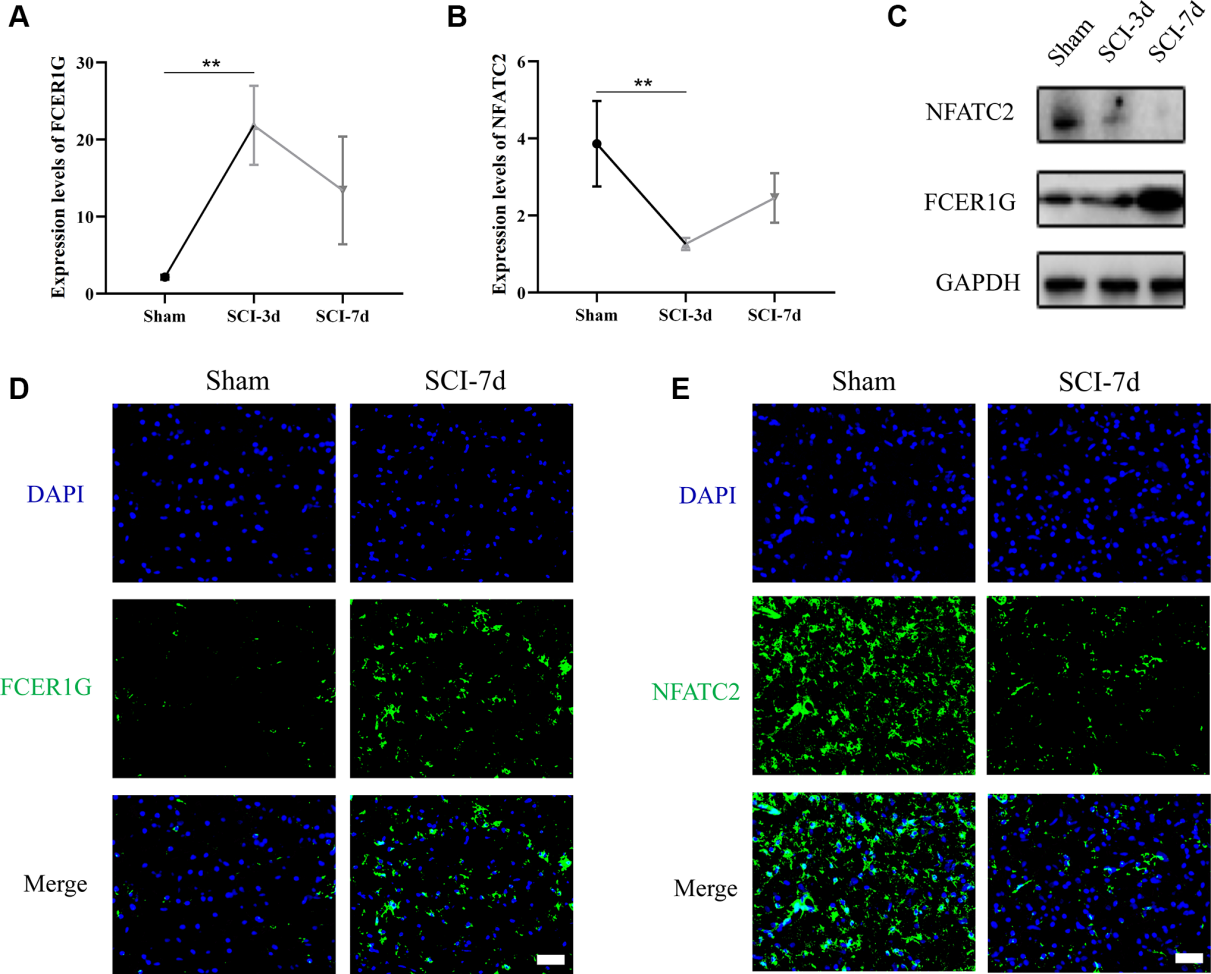
**Verification of the expression of Core IRGs via rat SCI model.** (**A**, **B**) Sequencing results of Core IRGs in SCI and sham group; (**C**) WB analysis demonstrating the expression of Core IRGs in the sham, SCI-3d and 7d group, respectively; (**D**, **E**) Immunofluorescence showed the expression of Core IRGs in sham and SCI-7d groups. Bar = 50 um; ^*^p < 0.05; ^**^p < 0.01; and ^***^p < 0.001.

Transcriptomic data from rat spinal cord tissues revealed that FCER1G expression was significantly higher in the SCI-d3 than in the sham group, while NFATC2 exhibited the opposite result (Figure 10A, 10B). Western blot (WB) analysis also demonstrated comparable trends (Figure 10C). Immunofluorescence (IF) analysis revealed that FCER1G protein expression in SCI-d7 was significantly above the sham group, while NFATC2 protein expression in SCI-d7 was visibly lower than that in the sham group (Figure 10D, 10E).

### Correlation and verification of core IRGs with immune cells

**Figure 11:**
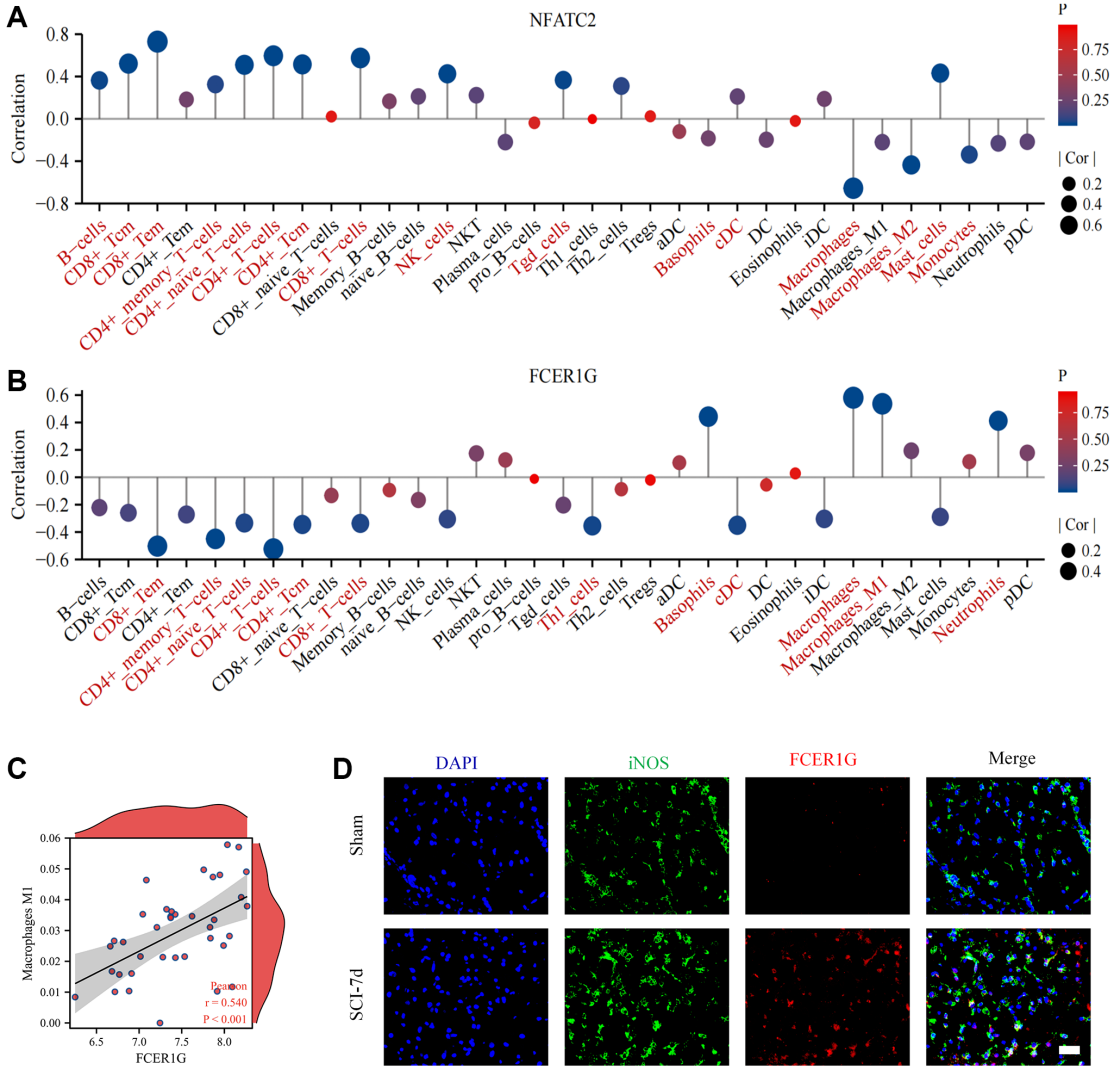
**Correlation of Core IRGs with immune cell infiltration.** (**A**, **B**) Core IRGs relevance to immune infiltrating cells in SCI; (**C**) Scatter plots of the correlation between FCER1G and macrophage M1; (**D**) Fluorescence analysis uncovered co-localization of FCER1G with iNOS.

We found that Core IRGs were intimately associated with multiple immune cells. NFATC2 was mainly positively correlated with a range of lymphoid cells and negatively correlated with myeloid cells (Figure 11A). In contrast, FCER1G was mostly positively correlated with a variety of myeloid cells, and negatively correlated with lymphoid cells (Figure 11B). Notably, FCER1G was positively linked to macrophage M1, which has been suggested to be essential immune cells in the development of pro-inflammatory effects of SCI (Figure 11C). Subsequently, IF analysis identified that FCER1G expression was colocalized with iNOS positive macrophage, which validated the relationship between FCER1G and macrophage M1(Figure 11D).

## DISCUSSION

Spinal cord injury is a severe CNS disorder that often leads to persistent organ dysfunction and permanent neurological deficits [1]. Despite the multiple methods that have been utilized to manage spinal cord injuries, including surgical decompression, pharmacotherapy, and physical rehabilitation, most patients still do not receive sufficient outcomes [4]. Consistent with the classic response to most organ injuries, SCI can trigger an intense immune inflammatory response, causing a rapid increase in circulating neutrophils, accompanied by immediate recruitment and infiltration of immune cells such as monocytes into the injured spinal cord site [15]. The immune response is a normal physiological consequence of the dramatic changes in the microenvironment following SCI, but this excessive response can entail further damage to spinal cord tissues, prevent repair and regeneration of spinal cord neurons, and ultimately worsen the clinical outcomes [6]. Therefore, a deeper understanding of the role of immune-related genes in the acute phase of SCI to suppress secondary damage will contribute to improving the development of novel therapies for SCI patients.

In this study, a total of 2067 DEGs were identified between SCI and HC groups. GSEA analysis identified inflammatory response, complement, and ROS to be more active in the SCI group than in the HC group. ROS is a single-electron reduction product of molecular oxygen involved in various physiological and pathological processes [19]. ROS have been proven to play an important part in the immune system, participating in the construction of innate and adaptive immunity and maintaining internal environmental homeostasis [20]. On the one hand, ROS is a key mediator of antigen presentation and immune cell activation. On the other hand, ROS can exert pro-inflammatory effects by controlling a wide range of signaling pathways [21]. Moreover, immune cells can release large amounts of ROS to exert immune-killing effects [22]. GO and KEGG analysis likewise revealed that these DEGs were associated with a broad range of immune-related pathways, including immune system process, immune response, Th1 and Th2 cell differentiation, and T cell receptor signaling pathway. These findings indicated that immune dysfunction played an essential role in the development and progression of SCI. Subsequently, the xCell algorithm uncovered a significantly more infiltrative abundance of neutrophils, macrophages M0, M1, and M2 in the SCI group than in the HC group, while a mass of lymphoid cells was markedly downregulated. These results were in agreement with previous studies. Following SCI, myeloid neutrophils rapidly enter the bloodstream and migrate and accumulate in the injured spinal cord through the endothelial barrier, exacerbating tissue inflammatory damage [23]. Activated neutrophils not only engulf localized necrotic tissue but also release elastase and reactive oxygen species to damage neurons and aggravate neurological dysfunction [15]. The infiltrating macrophages in the injured spinal cord consist of two sources, resident microglia and the peripheral source of myeloid macrophages. Macrophages as an element of the immune network possess a high level of plasticity. Under the stimulation of inflammatory factors, macrophages can polarize into M1 and M2 phenotypes, each exhibiting pro-inflammatory, and anti-inflammatory effects resulting in a dual immune response to SCI, as well as cytotoxic and neuroprotective effects [24–27]. Overall, these findings provide novel insights into the contribution of the immune response and immune microenvironment in SCI.

To further characterize the role of IRGs in SCI, WGCNA analysis was performed to screen for the closest modules related to the pathophysiology of SCI. WGCNA is an approach to analyzing the gene expression patterns of multiple samples by clustering functionally related genes and associating them with specific variables [28]. Using gene expression data from the peripheral blood of SCI patients, we constructed a gene co-expression network and found that the turquoise module was most positively associated with the pathogenesis of SCI. Subsequently, 51 major IRGs were screened through the intersection of IRGs, DEGs, and turquoise modules. We considered that these 51 IRGs were intimately associated with the regulation of the immune response in the pathological process after SCI. Based on the expression profiles of these IRGs, unsupervised clustering analysis divided SCI patients into two distinct immune modulation patterns. Anti-inflammatory immune cells including macrophages M0, M2, and Treg were up-regulated in Cluster 1, while pro-inflammatory immune cells such as CD8 T cells and NK cells were up-regulated in Cluster 2. Treg and macrophage M2 play an important role in the neuroinflammatory process after SCI, significantly suppressing the inflammatory response and reducing the secretion of inflammatory elements to promote the recovery of neurological function [26, 29]. In addition, ssGSEA analysis revealed that inflammation-promoting, T-cell co-stimulation, and cytolytic activity were more active in Cluster 2, which further validates the previous results. We believe that SCI patients in Cluster 2 had a more severe immune and inflammatory response, which results in poor distant functional recovery. Altogether, these findings provide new perspectives for the precise classification of SCI patients for personalized immunotherapy.

Considering the inaccuracy of a single algorithm, two machine learning algorithms and PPI analysis were applied to identify the most stable and reliable IRGs for the early diagnosis and treatment of SCI. The results identified a total of two IRGs (FCER1G and NFATC2), which exhibited accurate predictive performance for SCI patients. Notably, our study was conducted with transcriptomic data from peripheral blood samples, which already has gained clinical recognition due to the accessibility and feasibility of peripheral blood detection for the early diagnosis of SCI [30]. Subsequently, we validated the expression of these IRGs on an external validation cohort and rat SCI model. The results showed that FCER1G was remarkably upregulated after SCI, while NFATC2 was noticeably downregulated after SCI. These findings were consistent with the results of the bioinformatic analysis, further validating the reliability of these biomarkers. Moreover, we found that FCER1G and NFATC2 were tightly correlated with various immune infiltrating cells, especially FCER1G was positively correlated with macrophage M1. Immunofluorescence analysis was used to further validate the connection of FCER1G with macrophage M1 *in vivo*. Previous studies identified FCER1G was intimately involved in M1 pro-inflammatory cell infiltration in the cardiac immune microenvironment [31]. At the early stage of SCI, macrophage M1 continuously recruits into the injured area to swallow necrotic tissue, which is beneficial to the recovery of SCI to a certain extent [26]. However, the extensive infiltration of macrophage M1 in SCI tends to delay clearance, which activates and releases a substantial amount of inflammatory agents through NF-κB and STAT1 signaling pathways, causing an inflammatory cascade response to further exacerbate the tissue damage [32]. Thus, we suggested that the upregulation of FCER1G after SCI can exacerbate the neuroinflammatory response and cause further damage through macrophage M1 polarization. FCER1G is an important inflammation-related molecule that encodes the Fc receptor γ-chain (FcRγ). FcRY contains an immune receptor tyrosine-based activator motif (ITAM) which is a key signaling transducer of multiple pathways mediating autoimmunity and chronic inflammation [33]. Numerous studies have found that FCER1G is expressed on the surface of various immune cells, including neutrophils, macrophages, eosinophils, etc., [34, 35]. FCER1G has been shown to promote neuropathic pain after nerve injury via immune and defense pathways [36]. NFATC2 is a nuclear factor member of the activated T cell (NFAT) family with a central role in the induction of gene transcription in the immune response [37]. Upregulation of NFATC2 can activate T cells and induce the production of a large number of cytokines to participate in the immune process [38]. In the acute phase of SCI, we found that the expression of multiple T cells was significantly downregulated, which might be tightly related to the downregulation of NFATC2. Collectively, our results provided new biomarkers for the early diagnosis and immune infiltration of SCI which might be novel targets for the treatment of SCI.

There were several weaknesses in our study. First, this study was sourced from public databases and lacked corresponding clinical and prognostic information, resulting in no further assessment of prognosis. Secondly, the present study has a relatively small sample, so a larger sample is still needed to evaluate the predictive efficacy of Core IRGs. Third, the exact regulatory mechanisms between core IRGs and immune cells remain unclear, as extensive experiments remain necessary to elucidate these points in the future.

## CONCLUSIONS

In conclusion, we revealed for the first time the immune feature and microenvironmental aberrations in SCI. Moreover, we recognized two immune modulation patterns with different immune statuses in SCI patients. Besides, 2 Core IRGs were identified for early prediction of SCI and strongly correlated with immune cells. We also verified the colocalization of FCER1G with macrophage M1. These findings may offer new insights and targets to fully understand the impact of immune response in SCI.

## MATERIALS AND METHODS

### Data processing and sample collection

The microarray expression profile for SCI patients was acquired from GSE151371 in the GEO database. The data in GSE151371 were normalized before download and included the expression profiles of peripheral blood leukocytes from 38 SCI patients and 10 healthy controls [30]. Moreover, peripheral blood samples from 10 SCI patients and 8 HC were collected from Xi-Jing Hospital as independent validation analysis. This study was approved by the institutional review board of Xi-Jing Hospital, the Fourth Military Medical University, and all the patients provided signed informed consent. Demographic details of all subjects were presented in summary in Supplementary Table 1.

### Principal component analysis and screening of differentially expressed genes

PCA was used to discriminate the characteristics between SCI and HC groups. Then, the R “limma” package was adopted to filter DEGs, with |log_2_Fold |≥1 and *p*-value ≤ 0.05 set as the threshold.

### Functional enrichment analysis

R package “clusterProfiler” was applied to perform the KEGG pathway and GO terms enrichment analysis of DEGs. KEGG pathway is the collection of metabolic pathways to respond to molecular interactions and response networks, including metabolism, genetic information processing, environmental information processing, cellular processes, human disease, and drug pathways. GO terms include molecular functions, biological processes, and cellular components. In addition, GSEA analysis also identified hallmark gene sets between SCI and HC groups. *p* < 0.05 and FDR < 0.25 were considered as the criteria.

### Landscape of immune microenvironment

To evaluate the differences in immune infiltrating cells between SCI and HC groups, gene expression profiles were uploaded into xCELL (https://xcell.ucsf.edu/) to obtain the abundance of myeloid and lymphoid cells in peripheral blood [39]. In addition, the ssGSEA algorithm was performed to assess the immune function of each sample.

### Weight gene co-expression network analysis

WGCNA was performed to construct a co-expression network and identify functional modules [28]. To ensure the accuracy of the results, the top 25% absolute median difference genes were included and outlier genes and samples were removed. Then, the appropriate power for the weight parameter of the adjacent function is chosen by using the pickSoftThreshold function. Subsequently, the adjacency was transformed into a topological overlap matrix (TOM) with empirical soft threshold β = 6. To classify genes with similar expression profiles to identify modules, hierarchical clustering was performed based on 1-TOM and minimum gene size (*n* = 30). Modules with similar expression profiles were merged with the threshold value of 0.25.

### Identification of immune-related genes in SCI

A total of 1793 immune-related genes were obtained from the ImmPort database [40]. Following the determination of DEGs and key modules from WGCNA, Venn analysis was used to intersect the above three gene parts to obtain the major IRGs involved in the pathological progression of SCI.

### Classification of immune modulation patterns for SCI patients

Based on the expression profiles of these important IRGs, unsupervised clustering analysis was performed to determine the immune modulation patterns of SCI patients through the R package “Consensus Cluster Plus”. The cumulative distribution function (CDF), consensus matrix, and comparative change in area under the CDF curve were taken to ensure the optimal number of clusters.

### Screening of key IRGs for diagnosis of SCI patients

To improve the prediction accuracy, two machine learning algorithms and PPI analysis were employed to screen the candidate genes for SCI diagnosis. Random Forest is another type of machine learning that selects the smallest genetic set with the lowest error rate from massive data to recognize potential biomarkers [41]. LASSO is a linear regression machine learning based on gene expression profiles to filter key genes to help clinicians increase prediction accuracy [42]. The random forest algorithm monitors genes by the R package random forest, and the relative importance score >0.5 was used as the threshold value. The R package “glmnet” was used for the dimensionality reduction of the LASSO algorithm and the minimum lambda value was set as a threshold. Besides, the PPI network was established by the STRING database (version 11.0). Then, Cytoscape was utilized to build PPI visualization networks and screen hub genes by the Radiality algorithm in the Cytohubba [43]. The intersection of the results of the three algorithms was considered a Key IRGs. ROC curves were further used to evaluate the diagnostic effectiveness of Key IRGs.

### qRT-PCR

Total RNA from peripheral blood samples was extracted using TRIZOL methods. cDNA synthesis kit (Takara, China) was utilized to reverse transcribe the RNA. The TB Green Premux Ex Taq II (Tli RNaseH Plus) and Bio-Rad CFX96 real-time PCR system (Bio-Rad, United States) was employed for qRT-PCR. The internal control was GADPH. The relative expression was calculated based on the comparative Ct (2^−ΔΔCt^) method. The primer sequences of the Key IRGs were shown in Table 1.

**Table 1:** Primer sequences of the Core IRGs.

**Gene**	**Sequence (5′–3′)**
FCER1G	Forward: AGCAGTGGTCTTGCTCTTACT
	Reverse: TGCCTTTCGCACTTGGATCTT
NFATC2	Forward: CGATTCGGAGAGCCGGATAG
	Reverse: TGGGACGGAGTGATCTCGAT
GAPDH	Forward: GGAGCGAGATCCCTCCAAAAT
	Reverse: GGCTGTTGTCATACTTCTCATGG

### Establishment of SCI rat model and sequencing analysis

The Sprague Dawley rats were used to construct the SCI model, all animal experiments were approved by the Institutional Animal Care and Use Committee of the Fourth Military Medical University. The SCI model was based on our previously reported modified bilateral spinal cord clamping model: the rats were anesthetized by intraperitoneal injection of 10 g/L sodium pentobarbital, a median incision was made on the dorsal skin to expose the T10 spinous process, and a laminectomy was performed at T10 to expose the spinal cord. The spinal cord was then clamped by forceps for 40 s to cause SCI. In the Sham group, only the lamina was opened without damaging the spinal cord. Subsequently, based on our previously reported RNA sequencing results in rat spinal cord tissues, we validated the expression of Core IRGs [44].

### Western blotting analysis

Animals were perfused with saline at specific time points and approximately 2 cm segments were excised from the injured spinal cord. The RIPA lysis buffer (Beyotime, China) was used for extracting proteins. We used a BCA protein assay kit (Solarbio, China) for quantifying proteins. The proteins were then boiled, loaded, separated by electrophoresis, and transferred onto NC membranes. Using suitable primary antibodies, including anti-FECR1G (1:2000, DF13263, Affbiotech, China), anti-NFATC2 (1:2000, A3107, Abclonal, China), and anti-GAPDH (1:5000, 60004-1-Ig, Proteintech, China). the cell membranes were blocked with skim milk and incubated under 4°C overnight, followed by incubation with the secondary antibody (1:2000) for 1 h at room temperature and observation by ECL luminescence.

### Immunofluorescence staining

After 7 days of SCI, the rats were perfused with paraformaldehyde, dissected, and sectioned at the site of injury, paraformaldehyde-fixed, sucrose sunk and OCT embedded, followed by sectioning. Sections were treated with 0.3% TritonX-100 for 20 min and blocked with 5% BSA blocking solution at room temperature for 1 h. The appropriate primary antibody was added to the sections and incubated overnight at 4°C, including anti-FECR1G (1:100, DF13263, Affbiotech, China), anti-NFATC2 (1:100, A3107, Abclonal, China), and anti-iNOS (1:100, ab210823, Abcam, United Kingdom). Sections were incubated with a secondary antibody (1:200) at room temperature in the dark for 1 h. After washing with PBS for 10 min, cell nuclei were stained with DAPI.

### Statistics

Statistical analysis was conducted using the R 4.0.5 software, SPSS 21.0, and GraphPad Prism 8. We performed Willcoxon to determine differences between experimental groups and performed a one-way ANOVA to determine differences among the three groups. All differences among and between groups were considered to be statistically significant at *P* < 0.05 (^*^p < 0.05, ^**^p < 0.01, and ^***^p < 0.001).

### Data availability statement

The original contributions presented in the study are publicly available. The data of our sequencing request can be found here: National Center for Biotechnology Information (NCBI) BioProject database (PRJNA760277). The raw data supporting the conclusions of this article will be made available by the authors, without undue reservation.

## Supplementary Materials

Supplementary Table 1

## References

[1] Siracusa R, Paterniti I, Bruschetta G, Cordaro M, Impellizzeri D, Crupi R, Cuzzocrea S, Esposito E. The Association of Palmitoylethanolamide with Luteolin Decreases Autophagy in Spinal Cord Injury. Mol Neurobiol. 2016; 53:3783–92. 10.1007/s12035-015-9328-6 26143261 PMC4937098

[2] Spinal Cord Injury (SCI) 2016 Facts and Figures at a Glance. J Spinal Cord Med. 2016; 39:493–4. 10.1080/10790268.2016.1210925 27471859 PMC5102286

[3] Fehlings MG, Nguyen DH. Immunoglobulin G: a potential treatment to attenuate neuroinflammation following spinal cord injury. J Clin Immunol. 2010 (Suppl 1); 30:S109–12. 10.1007/s10875-010-9404-7 20437085 PMC2883090

[4] Wilson JR, Forgione N, Fehlings MG. Emerging therapies for acute traumatic spinal cord injury. CMAJ. 2013; 185:485–92. 10.1503/cmaj.121206 23228995 PMC3612151

[5] Wilson JR, Singh A, Craven C, Verrier MC, Drew B, Ahn H, Ford M, Fehlings MG. Early versus late surgery for traumatic spinal cord injury: the results of a prospective Canadian cohort study. Spinal Cord. 2012; 50:840–3. 10.1038/sc.2012.59 22565550

[6] Popovich P, McTigue D. Damage control in the nervous system: beware the immune system in spinal cord injury. Nat Med. 2009; 15:736–7. 10.1038/nm0709-736 19584863

[7] Schwab JM, Zhang Y, Kopp MA, Brommer B, Popovich PG. The paradox of chronic neuroinflammation, systemic immune suppression, autoimmunity after traumatic chronic spinal cord injury. Exp Neurol. 2014; 258:121–9. 10.1016/j.expneurol.2014.04.023 25017893 PMC4099970

[8] Filipp ME, Travis BJ, Henry SS, Idzikowski EC, Magnuson SA, Loh MY, Hellenbrand DJ, Hanna AS. Differences in neuroplasticity after spinal cord injury in varying animal models and humans. Neural Regen Res. 2019; 14:7–19. 10.4103/1673-5374.243694 30531063 PMC6263009

[9] Plemel JR, Wee Yong V, Stirling DP. Immune modulatory therapies for spinal cord injury--past, present and future. Exp Neurol. 2014; 258:91–104. 10.1016/j.expneurol.2014.01.025 25017890

[10] Hu X, Leak RK, Thomson AW, Yu F, Xia Y, Wechsler LR, Chen J. Promises and limitations of immune cell-based therapies in neurological disorders. Nat Rev Neurol. 2018; 14:559–68. 10.1038/s41582-018-0028-5 29925925 PMC6237550

[11] Kasinathan N, Vanathi MB, Subrahmanyam VM, Rao JV. A review on response of immune system in spinal cord injury and therapeutic agents useful in treatment. Curr Pharm Biotechnol. 2015; 16:26–34. 10.2174/1389201015666141031121338 25374028

[12] Gao TY, Huang FF, Xie YY, Wang WQ, Wang LD, Mu D, Cui Y, Wang B. Dynamic changes in the systemic immune responses of spinal cord injury model mice. Neural Regen Res. 2021; 16:382–7. 10.4103/1673-5374.290910 32859802 PMC7896203

[13] Bradbury EJ, Burnside ER. Moving beyond the glial scar for spinal cord repair. Nat Commun. 2019; 10:3879. 10.1038/s41467-019-11707-7 31462640 PMC6713740

[14] Yao Y, Xu J, Yu T, Chen Z, Xiao Z, Wang J, Hu Y, Wu Y, Zhu D. Flufenamic acid inhibits secondary hemorrhage and BSCB disruption after spinal cord injury. Theranostics. 2018; 8:4181–98. 10.7150/thno.25707 30128046 PMC6096396

[15] Feng Y, Peng Y, Jie J, Yang Y, Yang P. The immune microenvironment and tissue engineering strategies for spinal cord regeneration. Front Cell Neurosci. 2022; 16:969002. 10.3389/fncel.2022.969002 35990891 PMC9385973

[16] Chang Q, Hao Y, Wang Y, Zhou Y, Zhuo H, Zhao G. Bone marrow mesenchymal stem cell-derived exosomal microRNA-125a promotes M2 macrophage polarization in spinal cord injury by downregulating IRF5. Brain Res Bull. 2021; 170:199–210. 10.1016/j.brainresbull.2021.02.015 33609602

[17] Zhang Z, Liu X, Cheng D, Dang J, Mi Z, Shi Y, Wang L, Fan H. Unfolded Protein Response-Related Signature Associates With the Immune Microenvironment and Prognostic Prediction in Osteosarcoma. Front Genet. 2022; 13:911346. 10.3389/fgene.2022.911346 35754801 PMC9214238

[18] Zhang Z, Pan J, Cheng D, Shi Y, Wang L, Mi Z, Fu J, Tao H, Fan H. Expression of lactate-related signatures correlates with immunosuppressive microenvironment and prognostic prediction in ewing sarcoma. Front Genet. 2022; 13:965126. 10.3389/fgene.2022.965126 36092937 PMC9448906

[19] Mittler R. ROS Are Good. Trends Plant Sci. 2017; 22:11–9. 10.1016/j.tplants.2016.08.002 27666517

[20] Yang Z, Min Z, Yu B. Reactive oxygen species and immune regulation. Int Rev Immunol. 2020; 39:292–8. 10.1080/08830185.2020.1768251 32423322

[21] Yang Y, Bazhin AV, Werner J, Karakhanova S. Reactive oxygen species in the immune system. Int Rev Immunol. 2013; 32:249–70. 10.3109/08830185.2012.755176 23617726

[22] Herb M, Schramm M. Functions of ROS in Macrophages and Antimicrobial Immunity. Antioxidants (Basel). 2021; 10:313. 10.3390/antiox10020313 33669824 PMC7923022

[23] Ankeny DP, Popovich PG. Mechanisms and implications of adaptive immune responses after traumatic spinal cord injury. Neuroscience. 2009; 158:1112–21. 10.1016/j.neuroscience.2008.07.001 18674593 PMC2661571

[24] Kong X, Gao J. Macrophage polarization: a key event in the secondary phase of acute spinal cord injury. J Cell Mol Med. 2017; 21:941–54. 10.1111/jcmm.13034 27957787 PMC5387136

[25] Zhang Z, Sui R, Ge L, Xia D. Moxibustion exhibits therapeutic effects on spinal cord injury via modulating microbiota dysbiosis and macrophage polarization. Aging (Albany NY). 2022; 14:5800–11. 10.18632/aging.204184 35876627 PMC9365548

[26] Gensel JC, Zhang B. Macrophage activation and its role in repair and pathology after spinal cord injury. Brain Res. 2015; 1619:1–11. 10.1016/j.brainres.2014.12.045 25578260

[27] Zhang Z, Peng Y, Dang J, Liu X, Zhu D, Zhang Y, Shi Y, Fan H. Identification of key biomarkers related to epithelial-mesenchymal transition and immune infiltration in ameloblastoma using integrated bioinformatics analysis. Oral Dis. 2022. [Epub ahead of print]. 10.1111/odi.14173 35226761

[28] Langfelder P, Horvath S. WGCNA: an R package for weighted correlation network analysis. BMC Bioinformatics. 2008; 9:559. 10.1186/1471-2105-9-559 19114008 PMC2631488

[29] Lin W, Chen W, Liu W, Xu Z, Zhang L. Sirtuin4 suppresses the anti-neuroinflammatory activity of infiltrating regulatory T cells in the traumatically injured spinal cord. Immunology. 2019; 158:362–74. 10.1111/imm.13123 31559637 PMC6856927

[30] Kyritsis N, Torres-Espín A, Schupp PG, Huie JR, Chou A, Duong-Fernandez X, Thomas LH, Tsolinas RE, Hemmerle DD, Pascual LU, Singh V, Pan JZ, Talbott JF, et al. Diagnostic blood RNA profiles for human acute spinal cord injury. J Exp Med. 2021; 218:e20201795. 10.1084/jem.20201795 33512429 PMC7852457

[31] Zhao W, Wu T, Zhan J, Dong Z. Identification of the Immune Status of Hypertrophic Cardiomyopathy by Integrated Analysis of Bulk- and Single-Cell RNA Sequencing Data. Comput Math Methods Med. 2022; 2022:7153491. 10.1155/2022/7153491 36238494 PMC9553329

[32] Gaojian T, Dingfei Q, Linwei L, Xiaowei W, Zheng Z, Wei L, Tong Z, Benxiang N, Yanning Q, Wei Z, Jian C. Parthenolide promotes the repair of spinal cord injury by modulating M1/M2 polarization via the NF-κB and STAT 1/3 signaling pathway. Cell Death Discov. 2020; 6:97. 10.1038/s41420-020-00333-8 33083018 PMC7538575

[33] Yang R, Chen Z, Liang L, Ao S, Zhang J, Chang Z, Wang Z, Zhou Y, Duan X, Deng T. Fc Fragment of IgE Receptor Ig (FCER1G) acts as a key gene involved in cancer immune infiltration and tumour microenvironment. Immunology. 2023; 168:302–19. 10.1111/imm.13557 36054819

[34] Yu M, Eckart MR, Morgan AA, Mukai K, Butte AJ, Tsai M, Galli SJ. Identification of an IFN-γ/mast cell axis in a mouse model of chronic asthma. J Clin Invest. 2011; 121:3133–43. 10.1172/JCI43598 21737883 PMC3148724

[35] Podgórska D, Cieśla M, Kolarz B. FCER1G Gene Hypomethylation in Patients with Rheumatoid Arthritis. J Clin Med. 2022; 11:4664. 10.3390/jcm11164664 36012903 PMC9410058

[36] Wang J, Ma SH, Tao R, Xia LJ, Liu L, Jiang YH. Gene expression profile changes in rat dorsal horn after sciatic nerve injury. Neurol Res. 2017; 39:176–82. 10.1080/01616412.2016.1273590 28033741

[37] Gabriel CH, Gross F, Karl M, Stephanowitz H, Hennig AF, Weber M, Gryzik S, Bachmann I, Hecklau K, Wienands J, Schuchhardt J, Herzel H, Radbruch A, et al. Identification of Novel Nuclear Factor of Activated T Cell (NFAT)-associated Proteins in T Cells. J Biol Chem. 2016; 291:24172–87. 10.1074/jbc.M116.739326 27637333 PMC5104941

[38] Rengarajan J, Mowen KA, McBride KD, Smith ED, Singh H, Glimcher LH. Interferon regulatory factor 4 (IRF4) interacts with NFATc2 to modulate interleukin 4 gene expression. J Exp Med. 2002; 195:1003–12. 10.1084/jem.20011128 11956291 PMC2193700

[39] Aran D, Hu Z, Butte AJ. xCell: digitally portraying the tissue cellular heterogeneity landscape. Genome Biol. 2017; 18:220. 10.1186/s13059-017-1349-1 29141660 PMC5688663

[40] Bhattacharya S, Andorf S, Gomes L, Dunn P, Schaefer H, Pontius J, Berger P, Desborough V, Smith T, Campbell J, Thomson E, Monteiro R, Guimaraes P, et al. ImmPort: disseminating data to the public for the future of immunology. Immunol Res. 2014; 58:234–9. 10.1007/s12026-014-8516-1 24791905

[41] Díaz-Uriarte R, Alvarez de Andrés S. Gene selection and classification of microarray data using random forest. BMC Bioinformatics. 2006; 7:3. 10.1186/1471-2105-7-3 16398926 PMC1363357

[42] Wang H, Lengerich BJ, Aragam B, Xing EP. Precision Lasso: accounting for correlations and linear dependencies in high-dimensional genomic data. Bioinformatics. 2019; 35:1181–7. 10.1093/bioinformatics/bty750 30184048 PMC6449749

[43] Chin CH, Chen SH, Wu HH, Ho CW, Ko MT, Lin CY. cytoHubba: identifying hub objects and sub-networks from complex interactome. BMC Syst Biol. 2014 (Suppl 4); 8:S11. 10.1186/1752-0509-8-S4-S11 25521941 PMC4290687

[44] Wang X, Zhang Z, Zhu Z, Liang Z, Zuo X, Ju C, Song Z, Li X, Hu X, Wang Z. Photobiomodulation Promotes Repair Following Spinal Cord Injury by Regulating the Transformation of A1/A2 Reactive Astrocytes. Front Neurosci. 2021; 15:768262. 10.3389/fnins.2021.768262 34795557 PMC8593167

