# Supplementary material for: Comprehensive landscape of immune-based classifier related to early diagnosis and macrophage M1 in spinal cord injury
Source: Aging (Albany NY). 2023 Feb 23;15(4):1158–76. doi: 10.18632/aging.204548 (PMC10008498; doi:10.18632/aging.204548)
Supplement: Supplementary Table 1 [file aging-15-204548-s001.pdf]

SUPPLEMENTARY TABLE

Supplementary Table 1. Demographics and clinical data of all subjects.

Subjects	Gender	Age	BMI	Injured neurologic level
SCI patients				
1	M	41	22.4	T12
2	F	43	20.8	T12
3	M	61	24.5	T9
4	F	35	21.9	T12
5	M	55	23.2	T3
6	M	27	19.6	T11
7	M	43	22.3	T11
8	F	64	21.3	T12
9	M	36	23.4	T9
10	M	46	21.8	T12
Healthy control				
1	M	23	22.5	NA
2	M	47	23.8	NA
3	M	33	23.3	NA
4	F	25	21.1	NA
5	M	62	22.5	NA
6	M	32	21.7	NA
7	F	48	20.8	NA
8	M	58	20.6	NA
